# Molecular Identification of the Transient Species Mediating the Deactivation Dynamics of Solvated Guanosine and Deazaguanosine

**DOI:** 10.3390/molecules27030989

**Published:** 2022-02-01

**Authors:** Javier Ortín-Fernández, Jesús González-Vázquez, Lara Martínez-Fernández, Inés Corral

**Affiliations:** 1 Departamento de Química, Módulo 13, Universidad Autónoma de Madrid, 28049 Madrid, Spain; francisco.ortin@uam.es (J.O.-F.); jesus.gonzalezv@uam.es (J.G.-V.); 2 Institute for Advanced Research in Chemistry (IAdChem), Universidad Autónoma de Madrid, 28049 Madrid, Spain

**Keywords:** deoxyguanosine, deoxydeazaguanosine, solvent-solute interactions, ab initio calculations, molecular dynamics, deactivation mechanism, dual fluorescence

## Abstract

Small structural alterations of the purine/pyrimidine core have been related to important photophysical changes, such as the loss of photostability. Similarly to canonical nucleobases, solute-solvent interactions can lead to a change in the excited state lifetimes and/or to the interplay of different states in the photophysics of these modified nucleobases. To shed light on both effects, we here report a complete picture of the absorption spectra and excited state deactivation of deoxyguanosine and its closely related derivative, deoxydeazaguanosine, in water and methanol through the mapping of the excited state potential energy surfaces and molecular dynamics simulations at the TD-DFT level of theory. We show that the N by CH exchange in the imidazole ring of deoxyguanosine translates into a small red-shift of the bright states and slightly faster dynamics. In contrast, changing solvent from water to methanol implies the opposite, i.e., that the deactivation of both systems to the ground state is significantly hindered.

## 1. Introduction

The study of the photophysics of canonical and non-canonical nucleobases has captured the attention of the DNA community for its implications on the effect of light when interacting with the macromolecule or disclosing the origin of DNA photostability. Both experimental spectroscopy and molecular simulations, most of them performed in the gas phase, as a first approximation, have contributed to this knowledge. Today, it is well established that solvent effects are not innocuous, but instead have some impact in the optical properties and the photophysics of these systems. A paradigmatic example is that of 2-aminopurine, adenine’s positional isomer. In fact, microsolvation experiments have demonstrated that whilst isolated 2-aminopurine is essentially non-emissive, the coordination of a single water molecule can increase the excited state lifetime of this species up to 100 times, depending on the hydrogen bonding pattern established between the solvent molecule and the nucleobase [1].

Solvent solute interactions have also been demonstrated to have some impact on the absorption spectra and the potential energy landscapes of natural nucleobases [2,3,4,5,6,7,8,9,10,11,12,13,14,15,16,17,18,19,20]. For the five natural nucleobases, it has been generally observed that accounting for hydration by means of hybrid implicit explicit protocols, incorporating at least the solvent molecules from the first solvation shell, produces a strong destabilization of the S_nπ*_ states, simultaneous to the stabilization of the L_b_ state. The L_a_ state also experiences a stabilization in the case of the purines, adenine and guanine, and for the pyrimidines, uracil and thymine, but in the case of cytosine, a blue shift is observed for this state. These shifts can either result in an inversion of the state ordering or in a mixing of the excited states compared to gas phase.

In the following, we will discuss some of the general trends extracted by the studies investigating the impact of solvents in the potential energy surfaces of canonical nucleobases. In uracil, the incorporation of the water molecules of the first solvation shell does not significantly modify the energy of the most important internal conversion funnel, the ethylenic ππ*/S_0_ Conical Intersection (CI), although it has been demonstrated to favor the ππ*→nπ* transition [3] and to stabilize the internal conversion funnel involving C4 puckering [21]. Also in cytosine, introducing a single water molecule and a dielectric continuum was found to slightly stabilize the ethylenic ππ*/S_0_ CI [22], but other studies including additional solvent molecules revealed the presence of an energy barrier to access this CI [14]. For its part, in adenine and guanine, the incorporation of water molecules to the computational model produces a decrease in the slope of the L_a_ state, while it dissolves the existing gas phase minima connected to the L_b_ excited state, which directly crosses the L_a_ state [10,11].

Insight into the deactivation mechanism of guanine nucleotides was provided in 2010 by femtosecond fluorescence spectroscopic studies [23]. In this study, Miannay et al. observed a dual fluorescence signal (334 nm and a red tail spreading up to 700 nm) for this system and determined the existence of a correlation between the decrease in the excitation energy, the decrease in the fluorescence quantum yield, and the increase in the intensity in the least energetic emission band. This complex deactivation behavior was attributed to the trapping of part of the population in the planar potential energy landscape of the L_a_ state of solvated guanine (see above) that would account for the slowly appearing tail at the red edge of the main emission band at 334 nm. In fact, these authors report a multiexponential decay with lifetimes in the scale of the ps. More recent femtosecond broadband time-resolved fluorescence and transient absorption experiments for guanine nucleoside and nucleotide in water and methanol solvents [24] ascribe the non-standard dynamics of this system rather to the competition between two different nonradiative decay pathways, namely direct internal conversion from the L_a_ ππ* state and internal conversion mediated by the population of a charge transfer state of πσ* character, that would lead to a triphasic dynamics with τ_1_ and τ_2_ in the subps/ps time scale and τ_3_ in the ps time scale both in water and methanol. The authors base their hypothesis on the dependence of the dual emission on the solvent, and in the different electronic polarization of the two fluorescence bands. In 2021, the group of Crespo-Hernández revisited the deactivation dynamics of guanine nucleotide and investigated the photophysics of its isoelectronic analogue 7-deazaguanosine monophosphate, arising from the exchange of the N in position 7 of guanosine by a CH moiety, resorting to steady state and time-resolved spectroscopy and Density Functional Theory (DFT)/TD-DF calculations at the Franck–Condon (FC) region [25]. Interestingly, for the deazaguanosine analogue, the authors also observed a dual fluorescence and a similar deactivation dynamic to guanosine. For these two systems, they employed two and three component sequential kinetic models with lifetimes in methanol in the subps (τ_1_ and τ_2_) and ps (τ_3_) time scales, which they respectively assigned to internal conversion from L_b_ to L_a_, internal conversion from L_a_, and depopulation from a S_3_ πσ* intramolecular charge transfer state.

**Figure 1:**
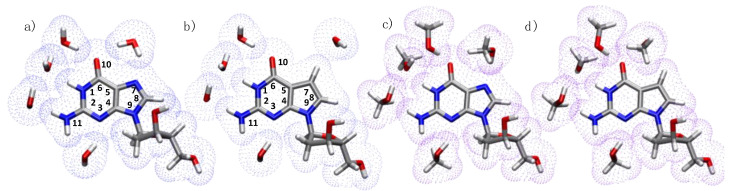
Systems considered in this study (**a**) dG.5H_2_O, (**b**) dAza.5H_2_O, (**c**) dG.5CH_3_OH, and (**d**) dAza.5CH_3_OH. Atom labeling shown in (**a**,**b**). In dots, the solvent accessible surface.

With the aim of shedding light into the decay mechanism of guanosine and its deaza analogue in a methanol and aqueous solvent environment (Figure 1), in this work, we present a computational study which, besides the characterization of the absorption spectrum, addresses the deactivation mechanism of these two systems by means of static calculations for the mapping of the excited state potential energy surfaces and molecular dynamics simulations.

## 2. Results and Discussion

All the photophysical simulations discussed below have been done on the most stable N1H-2-amino-6-oxo tautomer (see Supplementary Materials).

### 2.1. Absorption Spectra

#### 2.1.1. Deoxyguanosine

**Figure 2:**
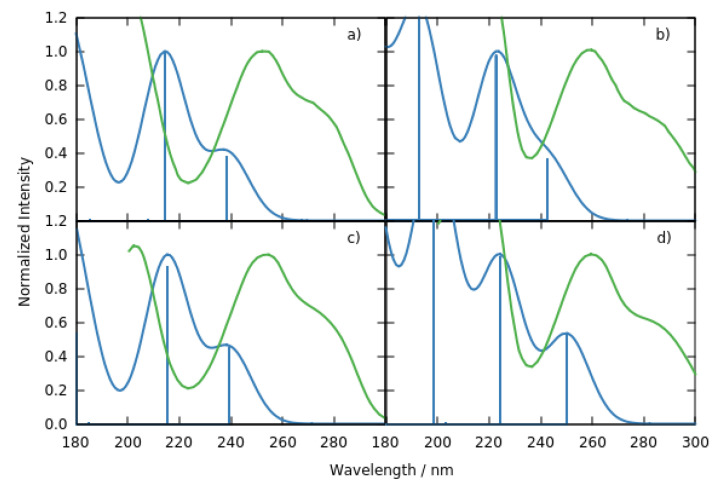
TD-M052X/6-31G*/PCM simulated absorption spectra (blue) for the deoxyguanosine.5H_2_O (**a**) and deoxyguanosine.5CH_3_OH (**b**) clusters and deoxydeazaguanosine.5H_2_O (**c**) and deoxydeazaguanosine.5CH_3_OH (**d**) clusters superimposed to the experimental absorption spectra. Reprinted from ref. [25] (green), with the permission of AIP Publishing. The height of each Gaussian is proportional to the oscillator strength of the corresponding excitation.

The absorption spectra and the vertical excitation energies in the range 180–300 nm of deoxyguanosine.5H_2_O (dG.5H_2_O) and deoxyguanosine.5CH_3_OH (dG.5CH_3_OH) clusters can be found in Figure 2a,b and Table 1, respectively. A one-to-one comparison with the experimental spectra recorded in phosphate buffer aqueous solution at pH 6.8 and in a mixture of methanol and water reveal that although the calculations are able to qualitatively reproduce the shape of the experimental spectra, they also shift the position of the absorption maxima to the blue by 0.7–0.8 eV [24,25], see Figure 2a. Similarly to previous theoretical works in the gas phase and considering implicit and explicit solvent solute interactions [13,25,26,27], our computational protocol predicts the L_a_ and L_b_ ππ* excited states as the lowest-lying. These ππ* states are followed by two nπ* excitations in the two solvents. Considering the typical gas phase → water solvent shift produced by five water molecules and a polarized continuum [11,13], there is an excellent agreement between our calculated energies for the L_b_ and first nπ* states and those calculated with CASPT2, whilst that of the L_a_ state is overestimated by 0.5 eV taking the CASPT2 calculations as benchmark [27]. Also interesting, our TD-DFT protocol predicts the L_b_ absorption to be more than twice as intense as the L_a_, in contrast with the CASPT2 results which predict both absorptions of the same intensity. In excellent agreement with the experimental findings [24], the exchange of water by methanol has very little impact on the vertical energies and oscillator strengths in the range of wavelengths considered, with the exception of the most stable nπ* state which shifts to the red by 0.2 eV and increases its intensity by ca. 30 times in methanol.

Beyond 180 nm, both water and methanol spectra are governed by strong ππ* transitions. When the spectrum is calculated considering methanol as solvent, however, some contribution of solvent → solute intermolecular charge transfer transitions to the high lying states S_8_ and S_9_ is observed.

#### 2.1.2. Deoxydeazaguanosine

The absorption spectra and vertical energies for the most stable excitations of the water and methanol deoxydeazaguanosine (dAza) clusters are shown in Figure 2c,d and Table 2. Also in this case, our simulations are able to reproduce the qualitative shape of the experimental spectra but overestimate the position of the absorption maxima by 0.7–0.8 eV [25]. The low energy region of the absorption spectra of dAza shares composition with that of dG, with the L_a_, L_b_, and a nπ* states absorbing at the long wavelength edge. The N by CH exchange in the imidazole only translates into a red-shift by 0.1–0.2 eV of the ππ* states, and in a blueshift by 0.3 eV for the nπ* state when the solvent is methanol. The first nπ* transition peaks at the same energy in the water spectra of dG and dAza. Our calculated vertical energies for these three states are in reasonable agreement (shifts ± 0.5 eV) with the TD-PBE0 values reported in the literature [25].

More important changes are observed when comparing dAza and dG for λ < 200 nm. In general, all the transitions in the dAza clusters are slightly shifted to higher energies compared to the dG clusters, and we also detect some character changes.

As for the case of the dG.5CH_3_OH cluster, for dAza.5CH_3_OH, we also observe some contribution of solvent → solute intermolecular charge transfer transitions to the high lying states.

At this point, it must be highlighted that in contrast to previous works [25], our calculations do not predict intramolecular charge transfer states or π→ Rydberg excited states below 150 nm, neither for the dG nor the dAza cluster. After the above analysis, we are in the position to assign the most recent experimental absorption spectra recorded by the group of Crespo-Hernández [25] and Kwok [24]. Despite the non-negligible shift produced by the neglect of explicit solvent solute interactions beyond the first solvation shell and the contribution of other geometries besides the FC one, similarly to other works, we assign the experimental maximum absorption bands and the shoulders at longer wavelengths of the formers to the ππ*(L_b_) and ππ*(L_a_) excitations.

### 2.2. Potential Energy Surfaces

#### 2.2.1. Deoxyguanosine

**Figure 3:**
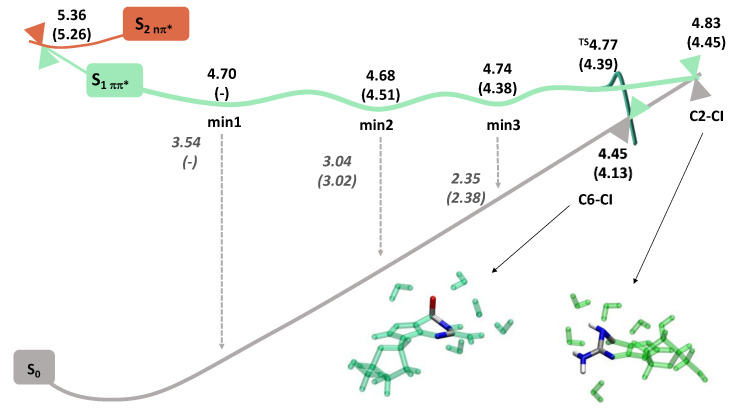
Potential Energy Surfaces for deoxyguanosine.5H_2_O and deoxydeazaguanosine.5H_2_O (numbers in parenthesis). Adiabatic (black) and emission (gray) energies in (eV). The geometries of C2-CI and C6-CI are shown for dG.

A schematic summary of the Potential Energy Surface (PES) of dG in water is depicted in Figure 3. The first two bright (ππ* L_a_ and ππ* L_b_) and dark (nπ*) excited states of dG.5H_2_O were optimized starting from the FC region. The first three states relax into the same minimum (min2 in Figure 3 and Figure S3) showing ππ* character, vertical emission energy (VEE) of ~3 eV, and still significant oscillator strength (0.07). Its main geometrical feature corresponds to an out-of-plane motion of the N1 and C2 (plus the N11H_2_ group) atoms, the dihedral angle d(C6-N1-N3-C2) being ~120 degrees (recall Figure 1 for atom numbering). Instead, the optimization of S_4_ nπ* state leads to another ππ* minimum (min3 in Figure 3) with significant lower emission energy (2.4 eV) and oscillator strength (0.03). In the search for additional minima with significant charge transfer character either to the sugar moiety or to the solvent as reported in other works [24,25], we optimized up to the S_10_ FC excited state. Such kind of minima were not located, but instead another ππ* minimum (min1 in Figure 3) in this case with larger VEE (3.5 eV) and f (0.12) and a S_2_ dark nπ* minimum were optimized. The latter is characterized by a large out-of-plane movement of the C6-O10 bond (Figure S3). All the ππ* minimum share the same characteristic dihedral but with different degrees of distortion, so we labeled them in decreasing order of their VEE: (ππ*)_min1_ = 3.5 > (ππ*)_min2_ = 3 > (ππ*)_min3_ = 2.3 eV. In terms of stability, i.e., adiabatic energy, the (ππ*)_min2_ is the most stable one (4.68 eV) followed by (ππ*)_min1_ (4.70 eV) and (ππ*)_min3_ (4.74 eV). In a subsequent step, we explored the non-radiative deactivation to the ground state from these minima. To do so, we performed a relaxed scan along the d(C6-N1-N3-**C2**) coordinate from the minimum with the lowest VEE, that is the smaller S_1_-S_0_ energy difference, which we assume to be closer to the corresponding S_1_/S_0_ funnel. We found that from (ππ*)_min3_, the system has to climb an upward potential energy profile to reach the S_1_/S_0_ CI, in the following C2-CI, that is higher in energy (+0.09 eV). It has previously been reported that in the condensed phase, an additional S_1_/S_0_ funnel involving a large distortion along the d(C2-N1-**C6**-O10) (C6-CI) coordinate competes with C2-CI in the deactivation [13]. For completeness, we, therefore, explored the connection between the (ππ*)_min3_ and the C6-CI. This CI (4.45 eV) is more stable than the minimum (4.74 eV) and a small transition state (+0.03 eV) separates these two stationary points. Both S_1_/S_0_ funnels are energetically accessible in agreement with the short-excited state lifetimes recorded experimentally for guanine in water solution (<2 ps) [24,25]. Most of these experiments, either based on transient absorption or fluorescence, have reported biphasic or multicomponent decays that have been related to different excited states showing different natures. However, an alternative explanation to the different lifetimes can be deactivation from the different regions of the planar PES.

**Figure 4:**
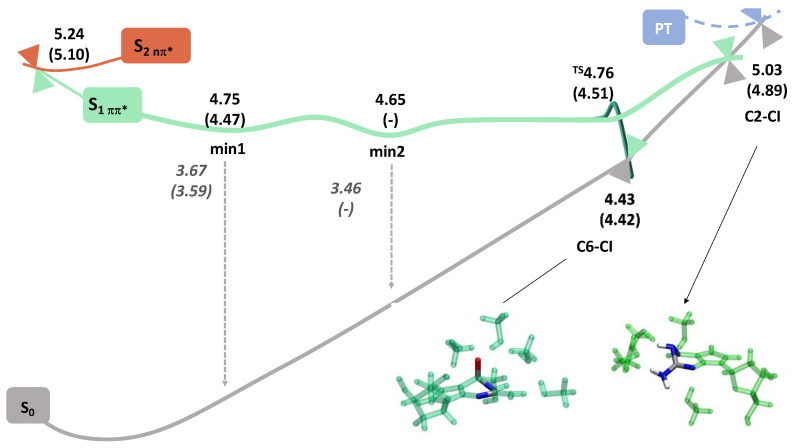
Potential Energy Surfaces for deoxyguanosine.5CH_3_OH and deoxydeazaguanosine.5CH_3_OH (numbers in parenthesis). Adiabatic (black) and emission (gray) energies in (eV). The geometries of C2-CI and C6-CI are shown for dAza.

Similar results were obtained when considering dG in methanol (Figure 4 and Figure S4). The geometry optimization of the first two bright (ππ* L_a_ and ππ* L_b_) states leads to the same ππ* minimum with VEE ~3.7 eV (min1 in Figure 4) and larger oscillator strength 0.13 compared to water in agreement with the FC picture. Analogously with dG in water, the optimization of the S_4_ nπ* state leads to another ππ* minimum with a lower emission energy (3.5 eV, min2 in Figure 4) and oscillator strength (0.09). The differences between min1 and min2 are very subtle in this case. Since these two minima have almost identical adiabatic energies (difference < 0.01 eV), they should be located very close in the PES. The optimization of S_3_ nπ* leads directly to a S_1_(nπ*)/S_0_ funnel. We have also optimized a S_2_ dark nπ* minimum starting from the higher in energy excited state at FC, and a third ππ* minimum (VEE = 3.5 eV and f = 0.10, not shown in Figure 4, min1bis), which is 0.1 eV more stable than min1. The labels employed attending to the VEE are the following in this case: (ππ*)_min1_ = 3.7 > (ππ*)_min1bis_ = 3.4 > (ππ*)_min2_ = 3.5 eV. Interestingly, the impact of the solvent on the non-radiative deactivation channels depends on the S_1_/S_0_ funnel considered. The C2-CI is very affected by switching the solvent from water to methanol: this crossing is higher in energy compared to its closest and most stable ππ* minimum, but in methanol the energy difference is one order of magnitude larger (+0.38 eV) compared to water (+0.09 eV), this channel being a priori energetically hampered. Instead, the access to the C6-CI is not dramatically altered, still being necessary to overcome a small transition state (+0.1 eV) comparable to the water situation. The dynamical behavior of dG in methanol is clearly multicomponent, still in agreement with the hypothesis of potential emission from different regions of the PES [24,25].

#### 2.2.2. Deoxydeazaguanosine

Despite one of the nπ* excited states in the dG spectra disappears in the dAza spectra due to the structural change in the imidazole ring, the global shape of the dAza PES in water (Figure 3) resembles the one described above for dG. The optimization of the first two bright states ends up in the same ππ* minimum with VEE ~3.0 eV and a modest oscillator strength 0.07. Also in the dAza case, the deactivation coordinate is the d(C6-N1-N3-C2) dihedral (Figure S5). The S_3_ nπ* state relaxes instead to a dark S_2_ nπ* minimum moving the C6-O10 bond significantly out of plane. A minimum in the same potential is found when the S_4_ ππ* is considered. No additional stationary points were located when optimizing the higher lying states S_5_-S_10_. However, taking as a guess the (ππ*)_min3_ geometry optimized for dG, we were able to locate a second minimum in dAza with VEE 2.4 eV and f = 0.05. In summary, we located two ππ* minima for dAza in water and we labeled them following the same nomenclature used above for dG: (ππ*)_min2_ = 3.0 > (ππ*)_min3_ = 2.4 eV. Further regions of the PES along the ground state deactivation were also characterized, the barriers towards the C2-CI (+0.07 eV) and C6-CI (+0.01eV) being even smaller than in dG, in agreement with the shorter lifetimes recorded experimentally. The absence of (ππ*)_min1_ in the dAza PES is the main difference with dG in water.

In methanol (Figure 4 and Figure S6), our results were practically identical but even simpler: only one bright minimum, (ππ*)_min1_, was located besides the S_2_ dark nπ* minimum starting from the S_3_ nπ* FC state. Interestingly, in this case, we located a minimum involving the transfer of one H atom from the solvent molecule towards the O10 atom in dAza. This minimum, in the following labeled as Proton Transfer (PT)_min_, was obtained from the optimization of the S_10_ FC state involving a clear CT from the solvent to the solute (recall Table 2). Its VEE is small 1.5 eV and the f is zero, pointing to a negligible contribution of this minimum to the fluorescence spectra (see below). Methanol has a great impact on the accessibility of C2-CI, that is significantly hampered (+0.4 eV), and to a lesser extent on C6-CI (+0.04 eV). Indeed, the experimental lifetime is 30% longer than in water [24,25].

### 2.3. Time Resolved Characterization

#### 2.3.1. Excited State Absorption Spectra

**Figure 5:**
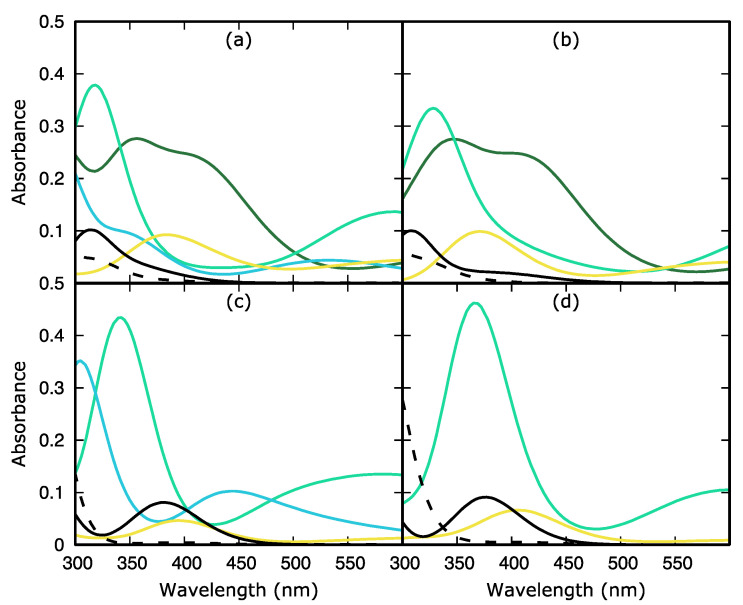
Excited state absorption spectra computed at all the optimized stationary points for deoxyguanosine.5H_2_O (**a**) and deoxyguanosine.5CH_3_OH (**b**) and deoxydeazaguanosine.5H_2_O (**c**) and deoxydeazaguanosine.5CH_3_OH (**d**) (ππ*)_min1_ in dark green, (ππ*)_min2_ in light green, (ππ*)_min3_ in cyan, (nπ*)_min1_ in yellow, C2-CI in solid black, and C6-CI in dashed black.

Transient absorption spectra (TAS) have been modeled by computing the excited-excited state vertical absorption energies and transition dipole moments (oscillator strengths) at each stationary point located along the PES exploration. Vibrationally hot ground state absorption was modeled considering the absorption of the S_0_ at the position of the S_1_/S_0_ conical intersections. The simulated spectra for dG and dAza in both water and methanol are shown in Figure 5.

Let us first discuss dG in water (Figure 5a) as it is the most complicated system, i.e., with the larger number of stationary points located. The absorption spectrum of (ππ*)_min1_ has an intense and very broad band in the range 350–450 nm. The dark S_2_ nπ* minimum also has a moderate absorption in this range of energies. In contrast, the other two minima absorb in different regions. Specifically, both have a main band ~300 nm and show less intense absorption in the 500–600 region, the absorption associated to (ππ*)_min2_ being much more important compared to (ππ*)_min3_. The overall shape of the excited state absorption spectrum in dG methanol (Figure 5b) is the same but for a noticeable decrease of intensity in red edge. The excited state absorptions of the minima present in dAza water ((ππ*)_min2_,(ππ*)_min3_ and nπ*) are very similar to the ones above described for dG water but for a uniform red-shift (in agreement with what was observed for the FC absorption) that is even more pronounced in methanol.

The experimental TAS [25] recorded for dG and dAza in buffer solution present three main absorption signals, in order of intensity, at ~350, ~420, and 570 nm whose absorption increases within the first 0.2–0.3 ps and then smoothly decays in tens of ps (see Figure S1). The main difference between dG and dAza is that the spectra of the former system are less structured, which could be attributed to the more complex PES, i.e., the larger number of stationary points located for this system. If all of them were populated along the decay of excited dG in water, a broad band of 300–700 nm would be expected, since their absorptions cover the complete range of wavelengths in the spectrum. The characteristic experimental bands in the TAS at ~350 and 570 nm could a priori be attributed to (ππ*)_min2_ since this minimum presents strong absorptions at this wavelengths and it is present in all the systems and solvents. To confirm such an assignment, the calculated individual spectra were linearly combined to fit the experimental spectra reported in [25], see Figure S1. Indeed, the fit (Table S1) gives up to 74% weights for this minimum, depending on the system and solvent, as part of the experimental TAS. On the other hand, the state responsible for the intermediate absorption at 420 nm is more difficult to identify. Minimum (ππ*)_min1_ could be a good candidate as it strongly contributes to the fitting for dG (37–72%); however, we were not able to locate this minimum for dAza for which (ππ*)_min3_ was found to importantly absorb there due to the red-shift previously mentioned, its contributions being in the range 30–47%. We, then, cannot discard any of these two minima as responsible for the absorption at 470 nm and possibly both minima are contributing in different extents to the TAS depending on the system and solvent considered (Table S1 and Figure S1).

#### 2.3.2. Dynamic Simulations and Fluorescence Spectra

**Figure 6:**
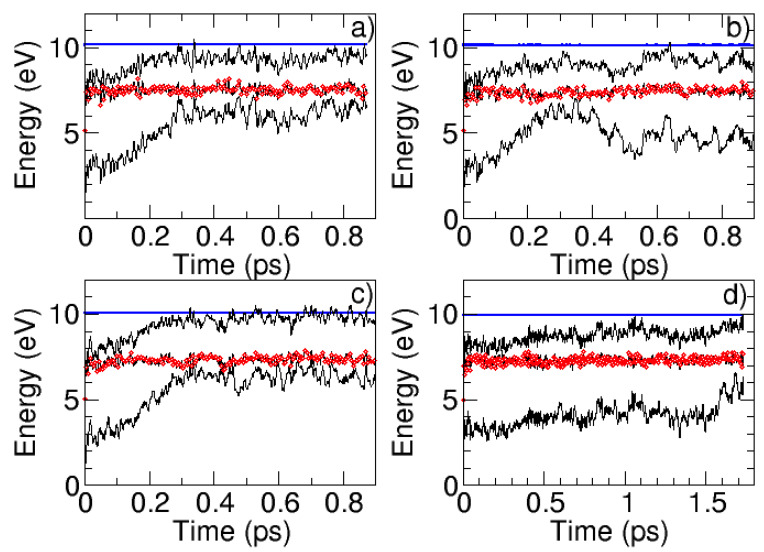
S_1_ adiabatic trajectories run for (**a**) deoxyguanosine.5H_2_O, (**b**) deoxyguanosine.5CH_3_OH, (**c**) deoxydeazaguanosine.5H_2_O, and (**d**) deoxydeazaguanosine.5CH_3_OH. The black lines correspond in increasing order of energy to the S_0_, S_1_, and S_2_ potentials. The red circles denote the potential visited by the system at specific times. The blue line depicts the total energy of the system, i.e., the sum of the potential and the kinetic energy.

The time-resolved information compiled from the comparison of the experimental and the theoretical transient absorption spectra was complemented with the analysis of several trajectories run on the S_1_ potential, which were also employed for the simulation of the emission spectra of these species. Figure 6 shows the details of the trajectories propagated.

All the trajectories, except for the dAza.5CH_3_OH cluster which was run for more time, were propagated for at least 1 ps, which is more than the time needed for the systems to evolve from the FC region to the S_1_/S_0_ degeneracy regions. The different panels in Figure 6 show the evolution in time of the potential energy for the first three electronic states (S_0_–S_2_). The red circles indicate the potential where the system is in each of the time steps. Since we are running adiabatic dynamics, the system is forced to remain in the S_1_ potential during the whole propagation.

A close inspection of the trajectories reveals for all the systems the proximity of the S_2_ and S_1_ potentials in the vicinity of the FC region as a result of the fast increase in the potential energy of the S_1_ due to the initial kinetic energy provided to the system. Moreover, interestingly, and despite the typical vibrational oscillations, in all the systems considered, the energy of the S_1_ potential remains almost constant during the total propagation time, denoting a quite planar potential in the pathway connecting the FC and the S_1_/S_0_ degeneracy regions, as found in the static analysis. This contrasts with the behavior of the S_0_ potential, which for all the systems shows an upward trend, favoring the evolution of the systems along the propagation towards the internal conversion funnel.

**Figure 7:**
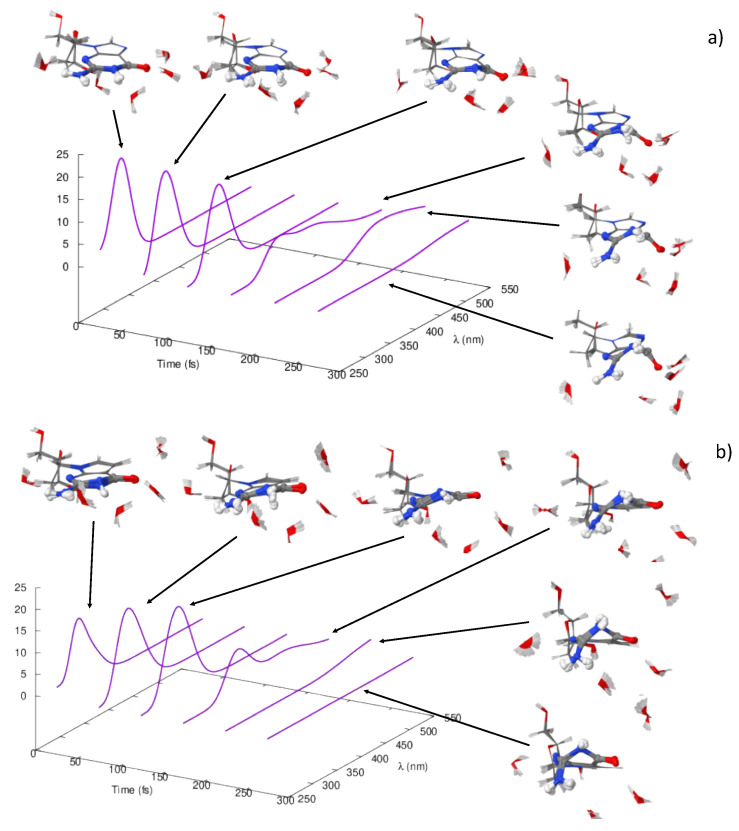
Time integrated fluorescence spectra in 50 fs time intervals (purple lines) for deoxyguanosine.5H_2_O (**a**), deoxydeazaguanosine.5H_2_O (**b**). Superposition of the geometries in the 50 fs intervals are also shown. Analogous spectra for the dynamics in methanol can be found in Figure S2.

From a structural point of view, all the systems start from a planar structure corresponding to the FC geometry (see Figure 7 and Figure S2). As the trajectories propagate, dG puckers at the N1 position. Simultaneously to that, the system experiences the out-of-plane deviation of the carbonyl group C6-O10 in the opposite direction, reminding very much of the C6-CI geometry and in agreement with the minimum energy pathway characterized through the static calculations.

The situation is slightly different for the dAza system (Figure 7b and Figure S2b). Similarly to dG, as the trajectories are propagated, the system initially experiences a puckering at the N1 position (Figure 7). In this case, however, the N1 puckering is accompanied by an out-of-plane distortion of the C2 center and the folding of the NH_2_ group in the opposite direction. For early propagation times, this folding is moderate as it occurs in the case of the different minima located in the static calculations of water and methanol solvated dAza, but it reaches an almost perpendicular orientation relative to the purine ring, as found for the C2-CI. Curiously, the deactivation mechanism revealed by the molecular dynamics simulations does not correspond to the most favorable route from an energetic point of view depicted by the static analysis, namely, the decay through the C6-CI funnel. Instead, dAza decays after the access to the C2-CI region which is only slightly destabilized when water solvent is considered but ca. 0.5 eV higher in energy when the simulations are performed in a methanol environment.

Unfortunately, the propagation time allowed for methanol-solvated deoxydeazaguanosine was not sufficient for the system to reach the S_1_/S_0_ conical intersection.

**Figure 8:**
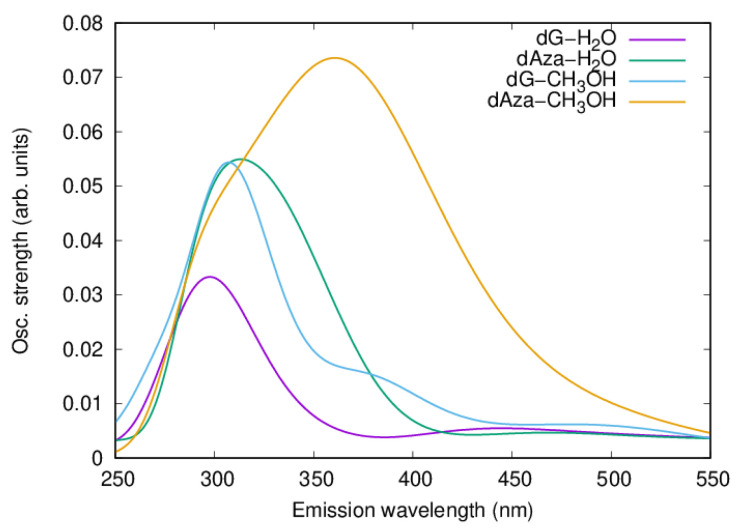
Simulated fluorescence spectra obtained from the time integration of the vertical emission of the structures registered along the trajectories (purple) deoxyguanosine.5H_2_O, (blue) deoxyguanosine.5CH_3_OH, (green) deoxydeazaguanosine.5H_2_O, and (yellow) deoxydeazaguanosine.5CH_3_OH.

Besides the structural evolution of the trajectories, Figure 7 shows the simulation of the emission spectra integrated in intervals of 50 fs. The total integration of this emission spectra is shown in Figure 8 and can be related with the fluorescence spectra.

Except for dAza.5CH_3_OH which presents a slower dynamic compared to the other systems studied, all the simulated spectra consist of two signals in the range between 250 and 550 nm. The lowest in energy and weakest energy bands are broad and cover the region between 400 and 550 nm in all the systems except dG.5CH_3_OH, where this band absorbs in the range between 350 and 550 nm. In turn, the maximum of the most intense band is centered in the 290–310 nm region for dG system whilst it peaks ca. 310–360 nm, in the case of dAza.

Considering the blue-shift inherent to our calculations, already observed for the absorption spectra, these results are in good agreement with the steady and time-resolved fluorescence spectra recorded by the groups of Crespo-Hernández and Kwok, which also register a dual fluorescence for these two systems in both solvents [24,25].

In fact, dG and dAza fluorescence spectra in methanol feature two emission maxima around 330 [25]/335 [24] and 415 [25]/520 nm [24] in the former and 310 and 430 nm in the latter [25]. In the case of water however, the emission spectra show a maximum at 334 nm followed by an unresolved tail at longer wavelengths [24].

A time-resolved analysis of the geometries originating these bands reveals that the strongest and most energetic band correlates with the first steps of the propagation, that is, with conformations close to that of the FC region, carrying a rather planar purine heterocycle. The lower energy and weaker band, however, so far registered for dG in both solvents and for dAza in water, only arises when longer propagation times are considered. This is the reason why this second band goes unnoticed in the spectrum of the dAza in methanol. Interestingly, this band would correlate with more distorted conformations, showing the out-of-plane distortion of different moieties, i.e., carbonyl group (C6) in dG and the C2 center in the case of dAza, which are close to the structures optimized for the conical intersections C6-CI and C2-CI.

## 3. Computational Details

Static Calculations. The ground (S_0_) and excited state (S_n_) minima of dG and dAza were optimized by means of Density Functional Theory (DFT) and its time-dependent version (TD-DFT). In particular, we selected M052X [28] and 6-31G(d) in view of the good performance of such functional on treating this kind of excited states [29]. The ground state absorption spectra were simulated by computing the vertical absorption energies (VAEs) and oscillator strengths on top of the S_0_ geometry with the same functional and basis set. For completeness, single point calculations were also done with the larger basis set (6-311++G(2df,2p), see Supplementary Materials, Table S2). Excited state absorption spectra were carried out following the same protocol but using the S_n_ geometries and the multiwfn program [30] to obtain the excited-excited state transition dipole moments to calculate the oscillator strength. The spectra shown in Figure 2 and Figure 5 were simulated by adding Gaussian functions at each VAE with a half width half maximum (HWHM) of 0.3 eV. All the calculations were done in solution by explicitly including 5 solvent molecules (accounting for the first solvation shell) plus an implicit model (accounting for the continuum, Polarizable Continuum Model, PCM) [31,32], using the Gaussian16 program [33]. TD-DFT degeneracy regions were estimated via relaxed scans along the coordinates that have been reported to connect the minima with the S_1_/S_0_ deactivation funnels.

Dynamic calculations. To follow the time evolution of the different species in the solvent environment, semiclassical dynamics in the first excited state were performed within the Born–Oppenheimer approximation and considering the time-dependent formalism. The trajectories were initialized in the S_0_ equilibrium geometry calculated as described above, with 5 eV of kinetic energy randomly distributed. The nuclear propagation was done using the Velocity Verlet algorithm with a time step of 0.5 fs. Concerning the electronic structure, energy and gradients were calculated using the TD-DFT approach within the Tamm–Dancoff approximation [34]. The long range corrected CAM-B3LYP [35] functional with the 6-31G* basis set was selected. As for the static calculations solvent solute interactions were described upon the incorporation of five implicit solvent molecules and a conductor-like polarizable continuum model [36] in the Linear Response part of the TD-DFT process to represent the rest of the solvent. These electronic structure calculations were performed using the ORCA program (5.0.1 version) [37,38] with the RIJCOSX [39,40] approximation using the embedded libxc [41] and libint2 [42] subroutines.

## 4. Conclusions

From the characterization of deoxyguanosine and its close derivative, deoxydeazaguanosine, excited states in water and methanol and the mapping of their potential energy surfaces and diabatic molecular dynamics simulations at the TD-DFT level of theory, we can conclude that:

In water,

(1) the main difference between dG and dAza absorption spectra is the occurrence of a small red-shift for the latter.
(2) The S_1_ potential energy surfaces of dG and dAza present a complex topology with several emissive ππ* minima, being more abundant in dG than in dAza.
(3) There are two deactivation funnels accessible in terms of energies, C6-CI and C2-CI, both being of more favored for dAza compared to dG, in agreement with slightly faster dynamics.

In contrast, methanol as a solvent has a significant impact on the photophysics of the canonical nucleobase dG and its derivative dAza. More specifically,

(1) it leads to a larger displacement (to the red) between the absorption spectra of dG and dAza.
(2) Methanol potential energy surfaces are simpler, with a small number of ππ* minima.
(3) The deactivation towards the ground state in methanol is significantly hindered from the energetic point of view. Indeed, the dynamics of dAza in this solvent are much slower, as experimentally observed.

There is a reasonable agreement between the simulated and the experimental emission spectra and TAS.

## Figures and Tables

**Table 1:** Vertical absorption energies calculated for the deoxyguanosine.5H_2_O and the deoxyguanosine.5CH_3_OH clusters in a water/methanol PCM continuum at the TD-M052X/6-31G* level of theory. Energies and wavelength in eV and nm.

Deoxyguanosine.5H_2_O		Deoxyguanosine.5CH_3_OH	
State (Character)	Energy/Wavelength (Osc. Strength)	State (Character)	Energy/Wavelength (Osc. Strength)
S_1_ ππ*(L_a_)	5.20/238 (0.1927)	S_1_ ππ*(L_a_)	5.18/239 (0.2159)
S_2_ ππ*(L_b_)	5.78/215 (0.4804)	S_2_ ππ*(L_b_)	5.76/215 (0.4512)
S_3_ nπ*	5.97/208 (0.0009)	S_3_ nπ*	5.76/215 (0.0311)
S_4_ nπ*	6.68/186 (0.0026)	S_4_ nπ*	6.70/185 (0.0031)
S_5_ ππ* (H-1→L; H→L+2)	6.89/180 (0.3295)	S_5_ ππ* (H-1→L; H→L+2)	6.88/180 (0.2603)
S_6_ ππ* (H→L+2; H-1→L;)	7.06/176 (0.1014)	S_6_ ππ* (H→L+2; H-1→L)	7.03/176 (0.1669)
S_7_ nπ*	7.17/173 (0.0004)	S_7_ nπ*	7.18/173 (0.0008)
S_8_ ππ* (H-3→L)	7.46/166 (0.2941)	S_8_ CT (n_meth_→L); ππ* (H-2→L)	7.31/170 (0.1679)
S_9_ ππ*	7.64/162 (0.0284)	S_9_ CT (n_meth_→L); ππ* (H-2→L)	7.46/166 (0.1360)
S_10_ ππ* (H-1→L+1)	7.68/161 (0.3403)	S_10_ mixed CT (n_meth_→L+2); ππ* (H-2→L+1)	7.63/162 (0.0457)

**Table 2:** Vertical absorption energies calculated for the deoxydeazaguanosine.5H_2_O and the deoxydeazaguanosine.5CH_3_OH clusters in a water/methanol PCM continuum at the TD-M052X/6-31G* level of theory. Energies and wavelength in eV and nm.

Deoxydeazaguanosine.5H_2_O		Deoxydeazaguanosine.5CH_3_OH	
State (Character)	Energy/Wavelength (Osc. Strength)	State (Character)	Energy/Wavelength (Osc. Strength)
S_1_ ππ*(L_a_)	5.11/243 (0.1505)	S_1_ ππ*(L_a_)	4.95/250 (0.1787)
S_2_ ππ*(L_b_)	5.57/223 (0.4054)	S_2_ ππ*(L_b_)	5.53/224 (0.3335)
S_3_ nπ*	5.94/209 (0.0006)	S_3_ nπ*	6.10/203 (0.0002)
S_4_ ππ* (H-1→L)	6.43/193 (0.5116)	S_4_ ππ* (H-1→L)	6.24/199 (0.5548)
S_5_ ππ* (H-1→L+1; H→L+2)	7.01/177 (0.0758)	S_5_ ππ* (H-1→L+1; H→L+2)	7.01/177 (0.0858)
S_6_ ππ* (H→L+2; H-1→L+1)	7.26/171 (0.2757)	S_6_ ππ* (H→L+2; H-1→L+1)	7.23/171 (0.2480)
S_7_ nπ*	7.36/168 (0.0135)	S_7_ nπ*; CT (n_meth_→L); ππ* (H-2→L)	7.28/170 (0.0983)
S_8_ nπ*	7.57/164 (0.0369)	S_8_ nπ*; CT (n_meth_→L); ππ* (H-2→L)	7.40/168 (0.0060)
S_9_ ππ* (H-2→L)	7.60/163 (0.1825)	S_9_ nπ*; (H-2→L)	7.46/166 (0.2056)
S_10_ ππ* (H-2→L+1); nπ*	7.92/157 (0.0503)	S_10_ CT (n_meth_→L)	7.63/162 (0.0060)

## Supplementary Materials

The following supporting information can be downloaded. Tautomer discussion References [43,44], Figure S1: Experimental Reference [25] and theoretical transient absorption spectra of deoxyguanosine and deoxydeazaguanosine in water and methanol.; Figure S2: Time integrated fluorescence spectra in 50 fs time intervals for dG.5CH_3_OH and dAza.CH_3_OH; Figures S3–S6: Geometries of the stationary points located along the PES of dG.5H_2_O, dG.CH_3_OH, dAza.5H_2_O and dAza.5CH_3_OH; Figures S7 and S8: Orbitals involved in the excited state of the stationary points in dG.5H_2_O and dAza.5CH_3_OH; Table S1: Percentage composition of the theoretical individual excited state absorption spectra to the transient absorption signals depicted in Figure S1; Table S2: Vertical absorption energies in a PCM continuum at the TD-M052X/ 6-311++G(2df,2p) level of theory.

## Sample Availability

It is a theoretical work, there are no samples.

## References

[1] Lobsiger S., Blaser S., Sinha R.K., Frey H.-M., Leutwyler S. (2014). Switching on the fluorescence of 2-aminopurine by site-selective microhydration. Nat. Chem..

[2] Gustavsson T., Bányász Á., Lazzarotto E., Markovitsi D., Scalmani G., Frisch M.J., Barone V., Improta R. (2006). Singlet Excited-State Behavior of Uracil and Thymine in Aqueous Solution:  A Combined Experimental and Computational Study of 11 Uracil Derivatives. J. Am. Chem. Soc..

[3] Santoro F., Barone V., Gustavsson T., Improta R. (2006). Solvent Effect on the Singlet Excited-State Lifetimes of Nucleic Acid Bases:  A Computational Study of 5-Fluorouracil and Uracil in Acetonitrile and Water. J. Am. Chem. Soc..

[4] Kistler K.A., Matsika S. (2009). Solvatochromic Shifts of Uracil and Cytosine Using a Combined Multireference Configuration Interaction/Molecular Dynamics Approach and the Fragment Molecular Orbital Method. J. Phys. Chem. A.

[5] Zazza C., Amadei A., Sanna N., Grandi A., Chillemi G., Di Nola A., D’Abramo M., Aschi M. (2006). Theoretical modeling of the valence UV spectra of 1,2,3-triazine and uracil in solution. Phys. Chem. Chem. Phys..

[6] Ludwig V., Coutinho K., Canuto S. (2007). A Monte Carlo-quantum mechanics study of the lowest n–π* and π–π* states of uracil in water. Phys. Chem. Chem. Phys..

[7] DeFusco A., Ivanic J., Schmidt M.W., Gordon M.S. (2011). Solvent-Induced Shifts in Electronic Spectra of Uracil. J. Phys. Chem. A.

[8] Höfener S., Gomes A.S.P., Visscher L. (2013). Solvatochromic shifts from coupled-cluster theory embedded in density functional theory. J. Chem. Phys..

[9] Colominas C., Luque F.J., Orozco M. (1996). Tautomerism and Protonation of Guanine and Cytosine. Implications in the Formation of Hydrogen-Bonded Complexes. J. Am. Chem. Soc..

[10] Gustavsson T., Sarkar N., Vayá I., Jiménez M.C., Markovitsi D., Improta R. (2013). A joint experimental/theoretical study of the ultrafast excited state deactivation of deoxyadenosine and 9-methyladenine in water and acetonitrile. Photochem. Photobiol. Sci..

[11] Karunakaran V., Kleinermanns K., Improta R., Kovalenko S.A. (2009). Photoinduced Dynamics of Guanosine Monophosphate in Water from Broad-Band Transient Absorption Spectroscopy and Quantum-Chemical Calculations. J. Am. Chem. Soc..

[12] Ludwig V., da Costa Z.M., do Amaral M.S., Borin A.C., Canuto S., Serrano-Andrés L. (2010). Photophysics and photostability of adenine in aqueous solution: A theoretical study. Chem. Phys. Lett..

[13] Improta R., Santoro F., Blancafort L. (2016). Quantum Mechanical Studies on the Photophysics and the Photochemistry of Nucleic Acids and Nucleobases. Chem. Rev..

[14] Martínez-Fernández L., Pepino A.J., Segarra-Martí J., Jovaišaitė J., Vaya I., Nenov A., Markovitsi D., Gustavsson T., Banyasz A., Garavelli M. (2017). Photophysics of Deoxycytidine and 5-Methyldeoxycytidine in Solution: A Comprehensive Picture by Quantum Mechanical Calculations and Femtosecond Fluorescence Spectroscopy. J. Am. Chem. Soc..

[15] Pepino A.J., Segarra-Martí J., Nenov A., Improta R., Garavelli M. (2017). Resolving Ultrafast Photoinduced Deactivations in Water-Solvated Pyrimidine Nucleosides. J. Phys. Chem. Lett..

[16] Szabla R., Kruse H., Šponer J., Góra R.W. (2017). Water–chromophore electron transfer determines the photochemistry of cytosine and cytidine. Phys. Chem. Chem. Phys..

[17] Improta R., Barone V., Barbatti M., Borin A.C., Ullrich S. (2015). Excited states behavior of nucleobases in solution: Insights from computational studies. Photoinduced Phenomena in Nucleic Acids I: Nucleobases in the Gas Phase and in Solvents.

[18] Barbatti M. (2014). Photorelaxation Induced by Water–Chromophore Electron Transfer. J. Am. Chem. Soc..

[19] Lischka H., Barbatti M., Siddique F., Das A., Aquino A.J.A. (2018). The effect of hydrogen bonding on the nonadiabatic dynamics of a thymine-water cluster. Chem. Phys..

[20] Kleinermanns K., Nachtigallová D., de Vries M.S. (2013). Excited state dynamics of DNA bases. Int. Rev. Phys. Chem..

[21] Minezawa N. (2014). Optimizing minimum free-energy crossing points in solution: Linear-response free energy/spin-flip density functional theory approach. J. Chem. Phys..

[22] Blancafort L., Migani A. (2007). Water effect on the excited-state decay paths of singlet excited cytosine. J. Photochem. Photobiol. A Chem..

[23] Miannay F.-A., Gustavsson T., Banyasz A., Markovitsi D. (2010). Excited-State Dynamics of dGMP Measured by Steady-State and Femtosecond Fluorescence Spectroscopy. J. Phys. Chem. A.

[24] Cheng C.C.-W., Ma C., Chan C.T.-L., Ho K.Y.-F., Kwok W.-M. (2013). The solvent effect and identification of a weakly emissive state in nonradiative dynamics of guanine nucleosides and nucleotides—A combined femtosecond broadband time-resolved fluorescence and transient absorption study. Photochem. Photobiol. Sci..

[25] Krul S.E., Hoehn S.J., Feierabend K.J., Crespo-Hernández C.E. (2021). Excited state dynamics of 7-deazaguanosine and guanosine 5′-monophosphate. J. Chem. Phys..

[26] Santoro F., Improta R., Fahleson T., Kauczor J., Norman P., Coriani S. (2014). Relative Stability of the La and Lb Excited States in Adenine and Guanine: Direct Evidence from TD-DFT Calculations of MCD Spectra. J. Phys. Chem. Lett..

[27] Serrano-Andrés L., Merchán M., Borin A.C. (2008). A Three-State Model for the Photophysics of Guanine. J. Am. Chem. Soc..

[28] Zhao Y., Schultz N.E., Truhlar D.G. (2006). Design of Density Functionals by Combining the Method of Constraint Satisfaction with Parametrization for Thermochemistry, Thermochemical Kinetics, and Noncovalent Interactions. J. Chem. Theory Comput..

[29] Zhao Y., Truhlar D.G. (2008). Density Functionals with Broad Applicability in Chemistry. Acc. Chem. Res..

[30] Lu T., Chen F. (2012). Multiwfn: A Multifunctional Wavefunction Analyze. J. Comput. Chem..

[31] Tomasi J., Mennucci B., Cammi R. (2005). Quantum Mechanical Continuum Solvation Models. Chem. Rev..

[32] Miertuš S., Scrocco E., Tomasi J. (1981). Electrostatic interaction of a solute with a continuum. A direct utilizaion of AB initio molecular potentials for the prevision of solvent effects. Chem. Phys..

[33] Frisch M.J., Trucks G.W., Schlegel H.B., Scuseria G.E., Robb M.A., Cheeseman J.R., Scalmani G., Barone V., Petersson G.A., Nakatsuji H. (2016). Gaussian 16 Rev. C.01.

[34] Hirata S., Head-Gordon M. (1999). Time-dependent density functional theory within the Tamm–Dancoff approximation. Chem. Phys. Lett..

[35] Yanai T., Tew D.P., Handy N.C. (2004). A new hybrid exchange–correlation functional using the Coulomb-attenuating method (CAM-B3LYP). Chem. Phys. Lett..

[36] Marenich A.V., Cramer C.J., Truhlar D.G. (2009). Universal Solvation Model Based on Solute Electron Density and on a Continuum Model of the Solvent Defined by the Bulk Dielectric Constant and Atomic Surface Tensions. J. Phys. Chem. B.

[37] Neese F. (2012). The ORCA Program System. WIREs Comput. Mol. Sci..

[38] Neese F. (2018). Software Update: The ORCA Program System, Version 4.0. WIREs Comput. Mol. Sci..

[39] Neese F., Wennmohs F., Hansen A., Becker U. (2009). Efficient, approximate and parallel Hartree–Fock and hybrid DFT calculations. A ‘chain-of-spheres’ algorithm for the Hartree–Fock exchange. Chem. Phys..

[40] Izsák R., Neese F. (2011). An overlap fitted chain of spheres exchange method. J. Chem. Phys..

[41] Lehtola S., Steigemann C., Oliveira M.J.T., Marques M.A.L. (2018). Recent developments in libxc—A comprehensive library of functionals for density functional theory. SoftwareX.

[42] Valeev E.F. (2014). A Library for the Evaluation of Molecular Integrals of Many-Body Operators over Gaussian Functions. http://libint.valeyev.net/.

[43] Gorb L., Leszczynski J. (1998). Intramolecular Proton Transfer in Mono- and Dihydrated Tautomers of Guanine:  An ab Initio Post Hartree−Fock Study. J. Am. Chem. Soc..

[44] Štoček J.R., Dračínský M. (2020). Tautomerism of Guanine Analogues. Biomolecules.

