# Supplementary material for: Molecular Identification of the Transient Species Mediating the Deactivation Dynamics of Solvated Guanosine and Deazaguanosine
Source: Molecules. 2022 Feb 1;27(3):989. doi: 10.3390/molecules27030989 (PMC8839017; doi:10.3390/molecules27030989)
Supplement: Supplementary file 1 [file molecules-27-00989-s001.zip › molecules-1561232-supplementary.pdf]

Supporting Information

Molecular identification of the transient species mediating the deactivation dynamics of solvated guanosine and deazaguanosine

Javier Ortín-Fernández,[1] Jesús González-Vázquez,[1,2] Lara Martínez-Fernández,[1,2*] Inés Corral[1,2*]

[1] Departamento de Química, Módulo 13, Universidad Autónoma de Madrid, 28049, Madrid, Spain.
[2] Institute for Advanced Research in Chemistry (IAdChem), Universidad Autónoma de Madrid, 28049, Madrid, Spain

1. Tautomers

The N1H-2-amino-6-oxo tautomer of the deoxynucleosides has been predicted by previous quantum mechanical calculations as the most stable. In fact, although the keto and the enol forms of guanine nucleobase are predicted to have similar stabilities in gas phase, introducing water-solute interactions (both via implicit and explicit models) importantly stabilizes the keto form.[1] For the deaza nucleoside, however, the 6-oxo 2-amino tautomer is already the most stable in the gas phase, lying ca. 9 $Kcalmol^{-1}$ below the enol and the N3H tautomers.[2]

2. Transient Absorption Fitting

The theoretical transient absorptions were calculated by linearly combining the excited absorption spectra calculated at the excited state minima to produce the best fitting of the experimental TAS upon minimizing an error function. A home made program was employed to this aim. Individual absorption spectra for the excited minima were calculated by overlapping gaussian functions with a HWHM of 0.3 eV on top of each excitation at the TD-DFT level of theory.

Table S1. Percentage composition of the theoretical individual excited state absorption spectra to the transient absorption signals depicted in Figure S1.

	$(\pi\pi^*)_{min1}$	$(\pi\pi^*)_{min2}$	$(\pi\pi^*)_{min3}$	$(n\pi^*)$
dG.5H_2O	37%	4%	47%	11%
dG.5CH_3OH	72%	28%	-	0%
dAza.5H_2O	-	68%	30%	2%
dAza.5CH_3OH	-	74%	-	26%

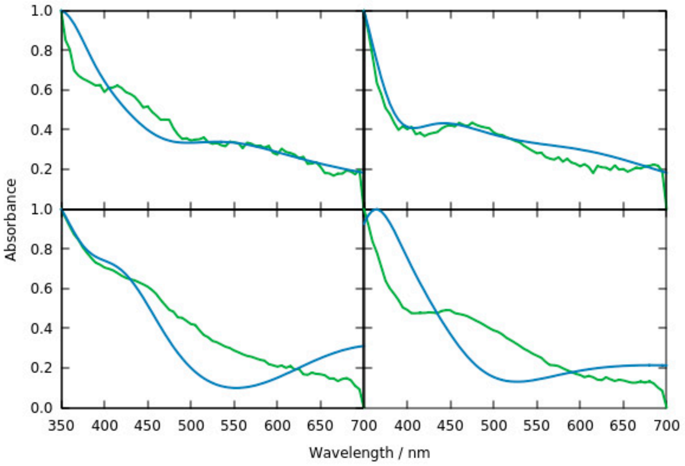

Figure S1. Experimental (green, τ ≈ 0.3 ps) and theoretical (blue) transient absorption spectra of deoxyguanosine and deoxydeazaguanosine in water (top left and top right respectively) and methanol (bottom left and bottom right respectively). Reprinted from [3], with the permission of AIP Publishing.

3. Additional Figures and Tables

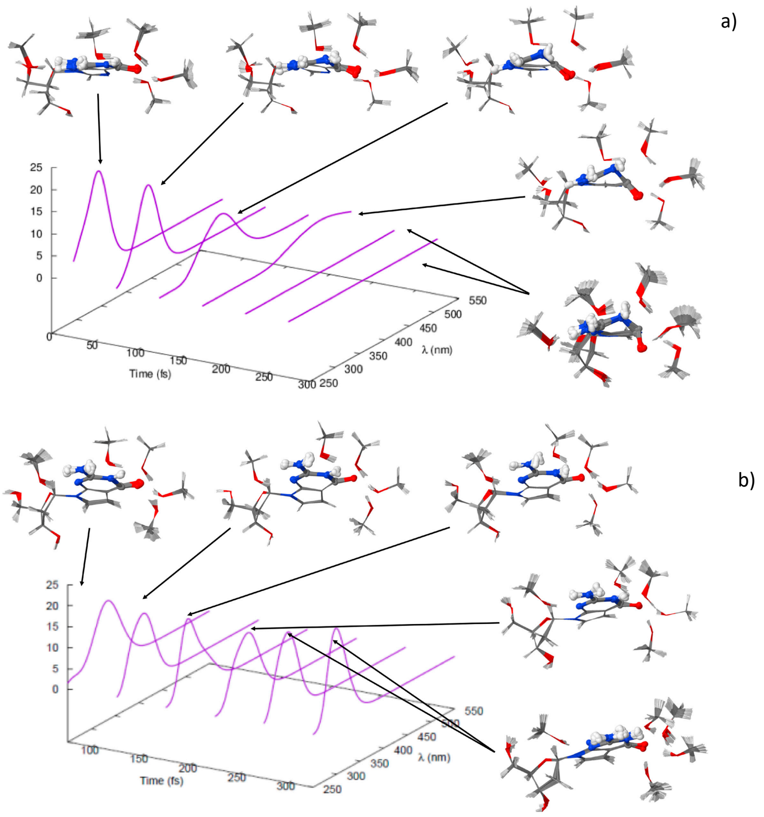

Figure S2. Time integrated fluorescence spectra in 50 fs time intervals (purple lines) for deoxyguanosine.5CH_3OH (a) deoxydeazaguanosine.CH_3OH (b).

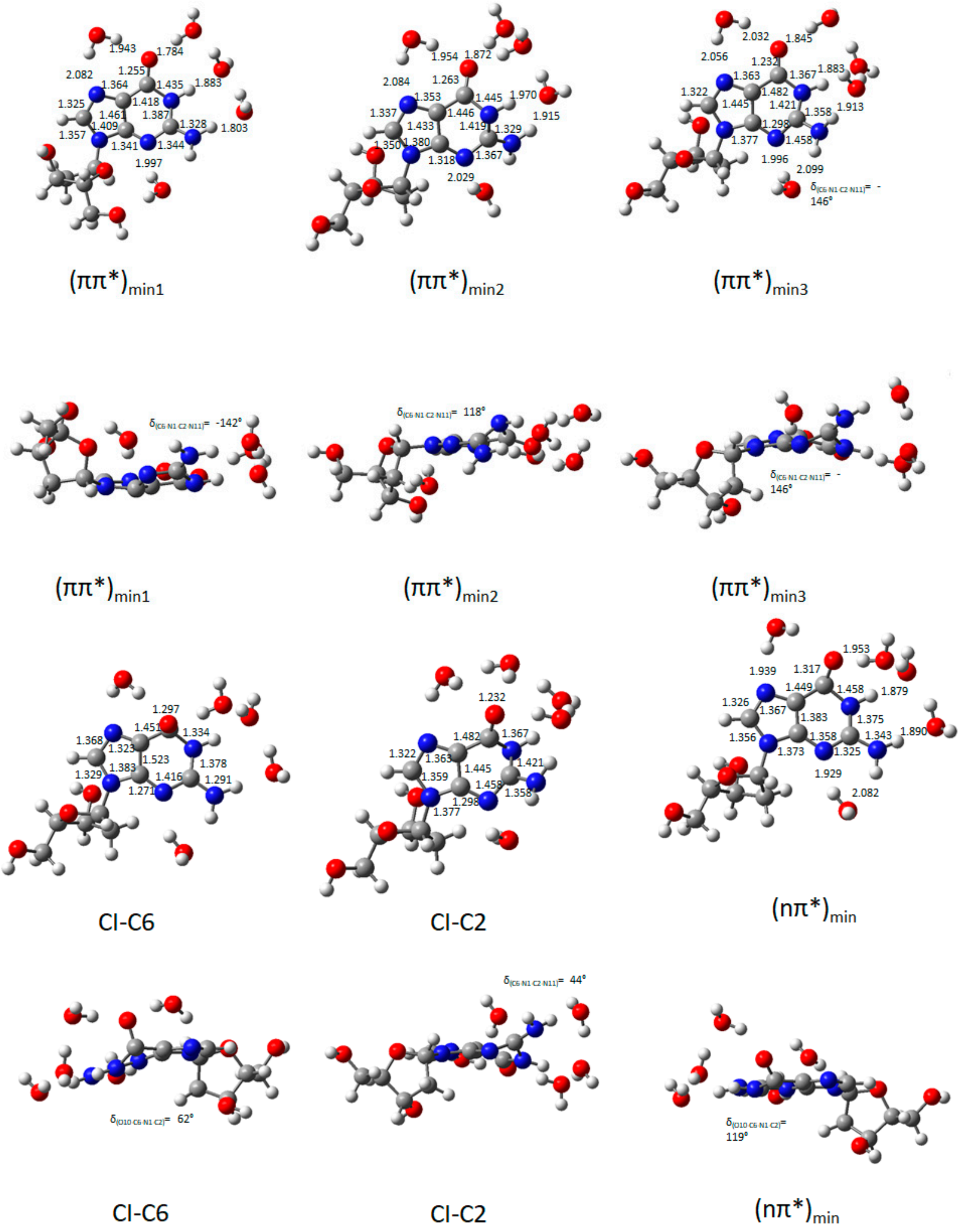

Figure S3: Geometries of the stationary points of deoxyguanosine.5H_2O PES.

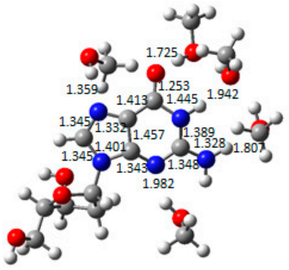

(ππ*)$_{min1}$

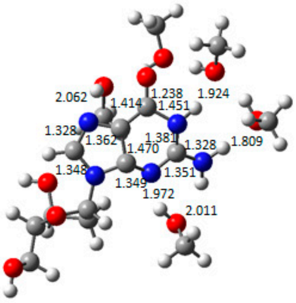

(ππ*)$_{min2}$

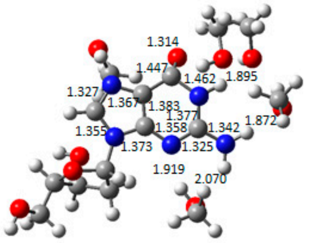

(nπ*)$_{min}$

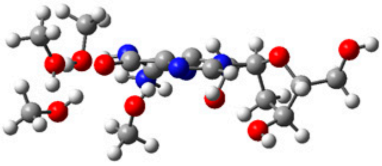

(ππ*)$_{min1}$

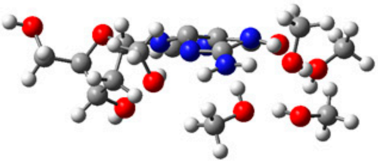

(ππ*)$_{min2}$

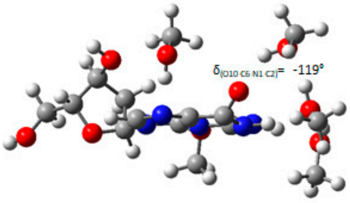

(nπ*)$_{min}$

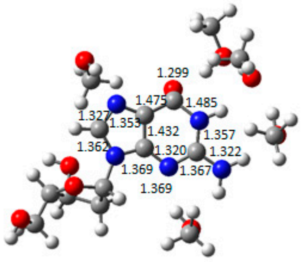

CI-C6

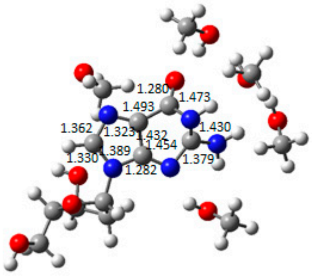

CI-C2

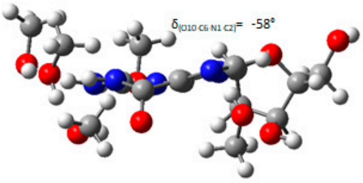

CI-C6

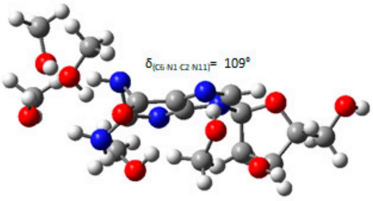

CI-C2

Figure S4: Geometries of the stationary points of deoxyguanosine.5CH_3OH PES.

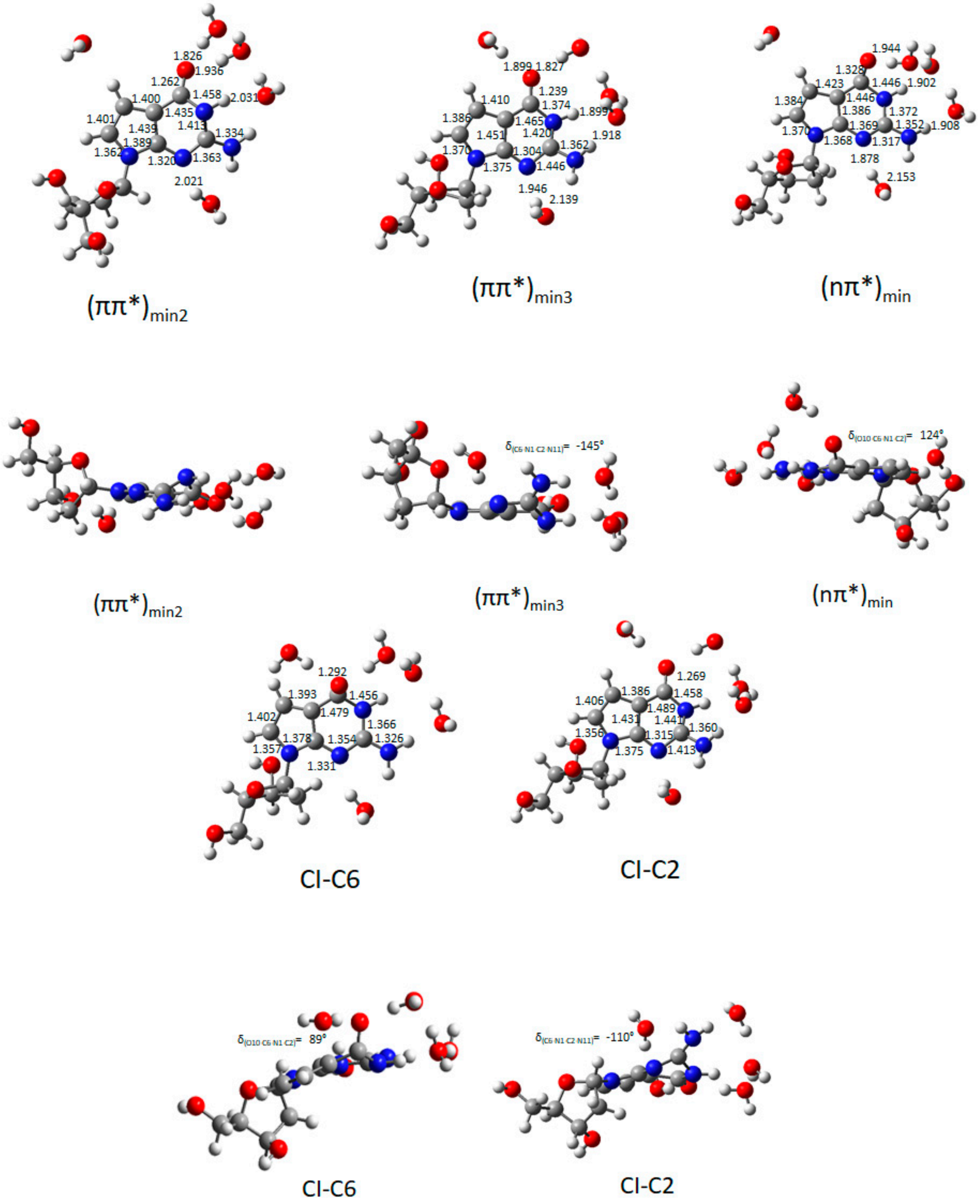

Figure S5: Geometries of the stationary points of deoxydeazaguanosine.5H_2O PES.

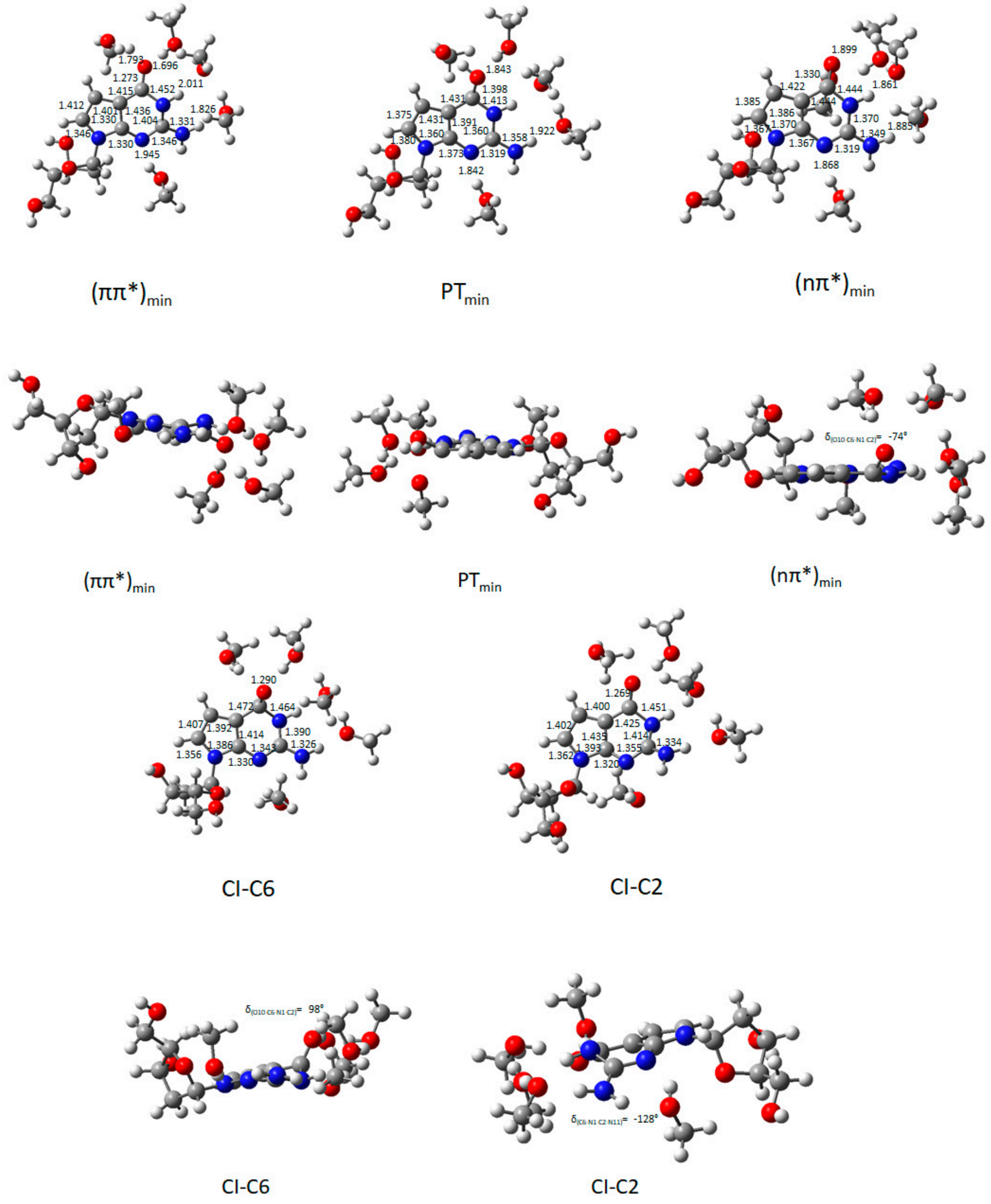

Figure S6: Geometries of the stationary points of deoxydeazaguanosine.5CH_3OH PES.

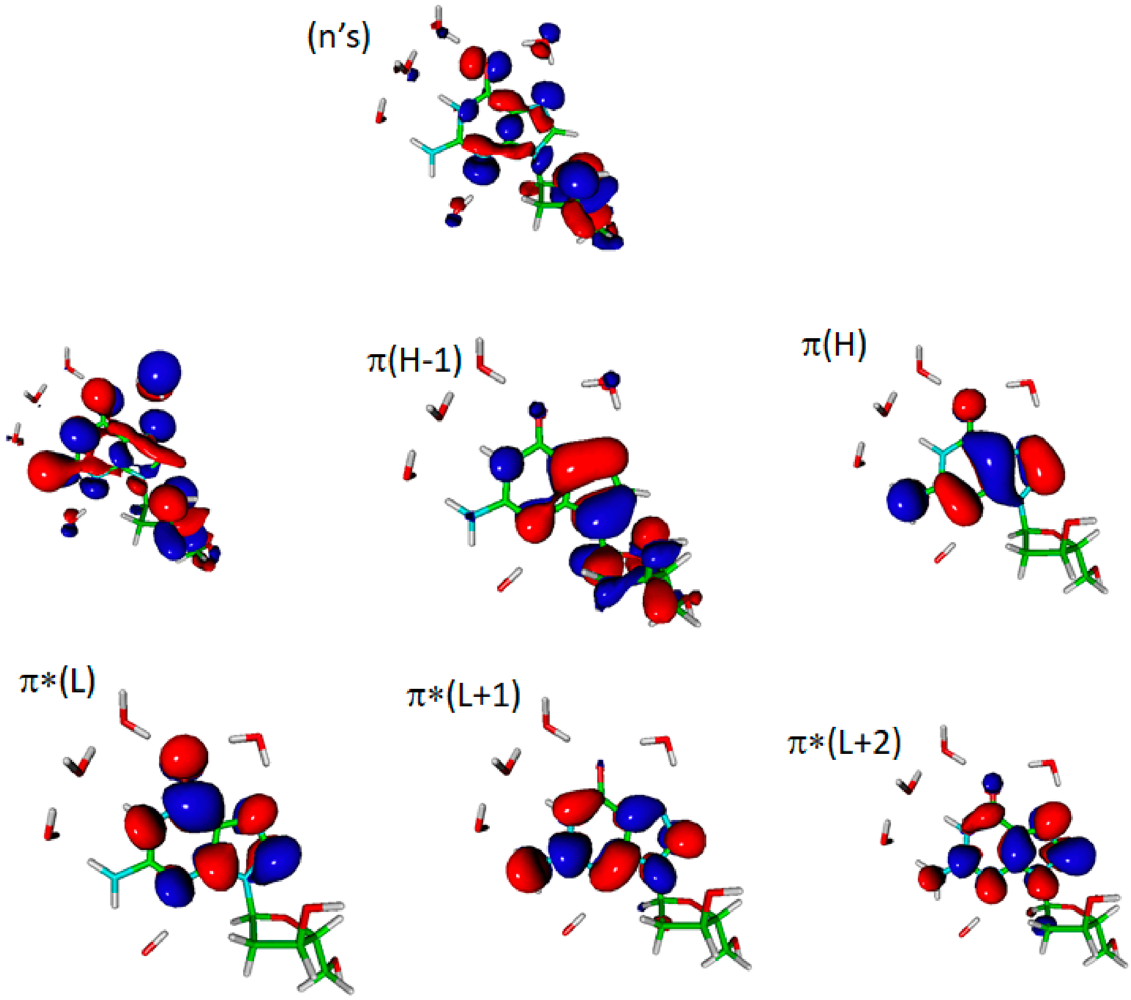

Figure S7: Orbitals involved in the excited states of deoxyguanosine.5H_2O.

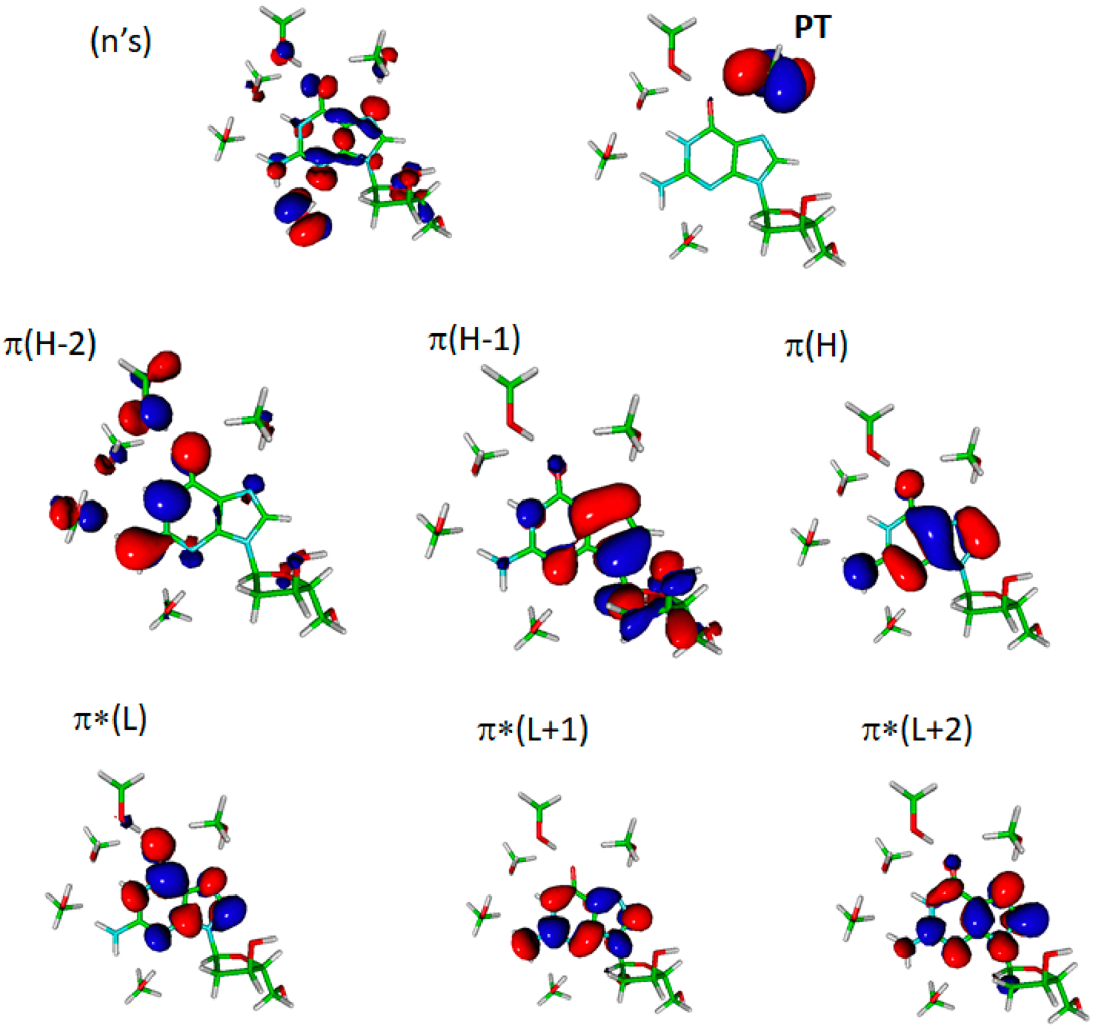

Figure S8: Orbitals involved in the excited state of the stationary points in deoxydeazaguanosine.5CH_3OH.

Table S2. Vertical absorption energies in a PCM continuum at the TD-M052X/ 6-311++G(2df,2p) level of theory. Energies and wavelength in eV and nm.

Deoxyguanosine.5H_2O		Deoxyguanosine.5CH_3OH	
**State**	**Energy/wavelength (osc. strength)**	**State**	**Energy/wavelength (osc. strength)**
S_1 ππ*	5.00/ 248 (0.2127)	S_1 ππ*	5.00/ 247 (0.2242)
S_2 ππ*	5.54/ 223 (0.4702)	S_2 ππ*	5.52/ 224 (0.4723)
S_3 nπ*	5.98/ 207 (0.0001)	S_3 nπ*	5.79/ 213 (0.0002)
S_4 ππ*	6.02/ 205 (0.0025)	S_4 ππ*	6.02/ 205 (0.0018)

Deoxydeazaguanosine.5H_2O		Deoxydeazaguanosine.5CH_3OH	
**State**	**Energy/wavelength (osc. strength)**	**State**	**Energy/wavelength (osc. strength)**
S_1 ππ*	4.90/252 (0.1632)	S_1 ππ*	4.80/258 (0.1831)
S_2 ππ*	5.29/234 (0.4249)	S_2 ππ*	5.29/234 (0.3773)
S_3 ππ*	5.56/209 (0.0071)	S_3 ππ*	5.70/217 (0.0031)
S_4 nπ*	5.98/207 (0.0003)	S_4 ππ*	6.05/204 (0.4594)

1. Gorb, L.; Leszczynski, J. Intramolecular Proton Transfer in Mono- and Dihydrated Tautomers of Guanine: An ab Initio Post Hartree−Fock Study. *Journal of the American Chemical Society* **1998**, *120*, 5024-5032, doi:10.1021/ja972017w.
2. Štoček, J.R.; Dračínský, M. Tautomerism of Guanine Analogues. *Biomolecules* **2020**, *10*, doi:10.3390/biom10020170.
3. Krul, S.E.; Hoehn, S.J.; Feierabend, K.J.; Crespo-Hernández, C.E. Excited state dynamics of 7-deazaguanosine and guanosine 5′ -monophosphate. *The Journal of Chemical Physics* **2021**, *154*, 075103, doi:10.1063/5.0038123.
